# Patients’ perception of communication at the interface between primary and secondary care: a cross-sectional survey in 34 countries

**DOI:** 10.1186/s12913-019-4848-9

**Published:** 2019-12-30

**Authors:** Giacomo Scaioli, Willemijn L. A. Schäfer, Wienke G. W. Boerma, Peter Spreeuwenberg, Michael van den Berg, François G. Schellevis, Peter P. Groenewegen

**Affiliations:** 1 Department of Public Health Sciences, University of Turin, Piazza Polonia, 94, 10126 Torino, Italy; 2 Department of Surgery, Northwestern University, Feinberg School of Medicine, 633 N. St Clair Street, Chicago, IL 60611 USA; 3 NIVEL (Netherlands Institute for Health Services Research), PO box 1568, 3500 BN Utrecht, The Netherlands; 4 Department of Public Health, Amsterdam Public Health Research Institute, PO Box 22660, 1100 DD Amsterdam, The Netherlands; 5 Department of General Practice and Elderly Care Medicine, Amsterdam Public Health Research Institute, VU University Medical Center, Van der Boechorststraat 7, 1081 BT Amsterdam, the Netherlands; 6 Department of Sociology, P.O. Box 80.115, 3508 TC Utrecht, The Netherlands; 7 Department of Human Geography, Utrecht University, P.O. Box 80.115, 3508 TC Utrecht, The Netherlands

**Keywords:** Patient views, Communication, Primary care, Secondary care, Referrals

## Abstract

**Background:**

Poor communication between general practitioners (GPs) and medical specialists can lead to poorer quality, and continuity, of care. Our study aims to assess patients’ perceptions of communication at the interface between primary and secondary care in 34 countries. It will analyse, too, whether this communication is associated with the organisation of primary care within a country, and with the characteristics of GPs and their patients.

**Methods:**

We conducted a cross-sectional survey among patients in 34 countries. Following a GP consultation, patients were asked two questions. Did they take to understand that their GP had informed medical specialists about their illness upon referral? And, secondly, did their GP know the results of the treatment by a medical specialist? We used multi-response logistic multilevel models to investigate the association of factors related to primary care, the GP, and the patient, with the patients’ perceptions of communication at the interface between primary and secondary care.

**Results:**

In total, 61,931 patients completed the questionnaire. We found large differences between countries, in both the patients’ perceptions of information shared by GPs with medical specialists, and the patients’ perceptions of the GPs’ awareness of the results of treatment by medical specialists. Patients whose GPs stated that they ‘seldom or never’ send referral letters, also less frequently perceived that their GP communicated with their medical specialists about their illness. Patients with GPs indicating they ‘seldom or never’ receive feedback from medical specialists, indicated less frequently that their GP would know the results of treatment by a medical specialist. Moreover, patients with a personal doctor perceived higher rates of communication in both directions at the interface between primary and secondary care.

**Conclusion:**

Generally, patients perceive there to be high rates of communication at the interface between primary and secondary care, but there are large differences between countries. Policies aimed at stimulating personal doctor arrangements could, potentially, enhance the continuity of care between primary and secondary care.

## Background

In modern health care systems, patients frequently visit both their general practitioner (GP) and multiple medical specialists. According to the ‘Health for All’ database, in 2013, each European citizen had, on average, an estimated seven primary care and/or ambulatory specialist care contacts [1]. Visits to multiple providers can result in the fragmentation of the patient’s care and the physician’s loss of important patient information. Furthermore, such loss of information can lead to delayed diagnoses, unnecessary repeated diagnostic examinations, and increased rates of adverse events [2–7]. It is important, in order to avoid this, that GPs and medical specialists communicate properly with their patients with regard to personal continuity of care. This communication, and indeed, cooperation too, should also extend to physicians working within the same care setting to ensure team continuity, and also to physicians from other care settings to ensure the continuity of care across various boundaries [8–12]. The latter is especially relevant when a patient is referred by a GP to a medical specialist. The transfer of patients’ information between a GP and a medical specialist is one of the essential steps to ensure a referral process of the highest quality [13]. Unfortunately, such communication may occur inconsistently, with large differences among countries (Scaioli G, Schäfer W, Boerma W , Spreeuwenberg P, Schellevis FG, Groenewegen PP. Communication between general practitioners and medical specialists in the referral process: a cross sectional survey in 34 countries, submitted).

Previous studies demonstrated that all actors in the referral process, the GPs, medical specialists, and patients, are aware of the importance of good communication between GPs and medical specialists [3, 7]. Patients understand that better communication at the interface between primary and secondary care results in better health outcomes for them. In addition, they do not want to have to repeat their medical history to every physician whom they consult [7, 14–16]. Moreover, patient satisfaction with the preparatory information given by their referring physician was associated positively with a patient’s trust in their referring physician [17]. In summary, to ease the referral process, it is important that GPs and medical specialists communicate properly with each other and that patients are aware of this communication [4, 18, 19]. Patients have a unique position in assessing communication between GPs and medical specialists as they are present in both the GP’s and the medical specialist’s office during the consultation [4]. It is, therefore, important that communication between GPs and medical specialists is measured from both the clinician’s and the patient’s perspective [20].

**Fig. 1:**
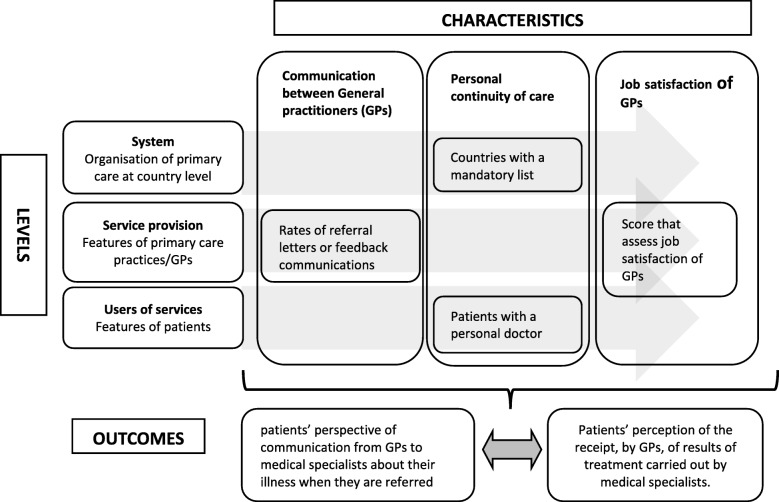
Visual model: features that potentially influence patients’ perspective of communication between GPs and medical specialists

Previous studies identified factors which were associated, potentially, with the patient’s perception of communication at the interface between primary and secondary care [4, 11]. However, these studies were performed in single countries while the organisation of primary care differs greatly among countries. These differences, therefore, might affect the attitudes and practices of GPs and consequently influence patients’ experiences, such as the patients’ perceptions of communication between GPs and medical specialists [21–23]. Using survey data on patients from 34 countries, our study analyses whether differences between countries in the organisation of primary care are related to patients perceived rates of communication at the interface between primary and secondary care. Further, we investigate whether the characteristics of GPs, primary care practices, and patients, are associated with the perceived communication, independent of the national structure of primary care. We hypothesise that the following factors are associated with higher rates of patients’ perceived communication at this interface:

1. *High rates of communication between GPs and medical specialists as reported by GPs*. Patients usually carry paper referral letters and feedback from the medical specialist to their GP. Therefore, patients are more likely to be aware of when a GP communicates with a medical specialist. We hypothesise that there is a positive correlation between GPs’ and patients’ perceived rates of communication at the interface between primary and secondary care.
2. *High rates of personal continuity of care*. Personal continuity of care is defined as “having a personal care provider in every separate care setting who knows and follows the patient” [9]. Studies conducted in the United States and Canada demonstrated that patients who usually consult the same GP perceive better coordination and communication between primary and secondary care [4, 11]. Always consulting the same physician, and/or primary care practice, may give the patients the feeling that their GP knows them well, and, therefore, the confidence that their GP and medical specialists are exchanging communication.
3. *Higher GP job satisfaction*. Several studies demonstrated that job satisfaction among physicians is associated positively with their quality of care [24–26]. Low job satisfaction among physicians leads to reduced workplace productivity and efficiency, increased absenteeism, less practice revenue, and reduced time with patients [24]. A recent study, demonstrated that a higher subjective workload among GPs, defined as feelings of being overworked, low job satisfaction and high job stress, [27] is correlated with worse patients’ experiences of primary health care services (Schäfer WL, Van den Berg M, Groenewegen PP. The association between the workload of family physicians and patient experiences with care, submitted)
4. *We also hypothesise that there is a correlation between the patients’ perception of communication from GPs to medical specialists and that from medical specialists to GPs*. Our hypothesis is that when a patient is aware of the GP sending letters to a medical specialist upon referral, this will enhance the patients’ perceptions about the GP being informed by the medical specialist after treatment. Variations among countries, in the communication perceived by the patient at the interface between primary and secondary care, are influenced by the above-mentioned features (See Fig. 1 for a graphical presentation of the relationships). The results of this study might be useful for both GPs and policy makers, in order to implement strategies to improve awareness among patients about communication between GPs and medical specialists.

## Methods

To analyse patients’ perceptions of communication at the interface between primary and secondary care in 34 countries, we used data from the QUALICOPC study (Quality and Costs of Primary Care in Europe). In this study, patients in 31 European countries (Austria, Belgium, Bulgaria, Cyprus, Czech Republic, Denmark, England, Estonia, Finland, Germany, Greece, Hungary, Iceland, Ireland, Italy, Latvia, Lithuania, Luxembourg, Malta, the Netherlands, Norway, Poland, Portugal, Romania, Slovakia, Slovenia, Spain, Sweden, Switzerland, North Macedonia, Turkey), plus in Australia, Canada, and New Zealand, were surveyed between October 2011 and December 2013. The study is co-funded by the European Commission, and, therefore, initially included only European countries. Research teams from Australia, Canada, and New Zealand joined the study on their own initiative, and with their own funding. This was due to their interest in an international comparison of primary care in their country. In each country, a target number of 200 GPs were asked to participate by filling out a questionnaire and allowing their patients to be interviewed. Only one GP could participate per practice. Fieldworkers were instructed to invite, consecutively, patients, aged 18 years or older, in the waiting room of each GP practice participating. Patients, who had had a face-to-face consultation with the GP participating, were invited to complete a questionnaire, anonymously, until nine patient questionnaires were collected in each practice. A target response of 2000 patients was required for each country, with the exception of Cyprus, Iceland, Luxembourg and Malta (target *N* = 800 patients). In Turkey, Spain, Belgium, and Canada, larger samples were taken in order to enable comparisons between regions. A total of 61,931 patients filled out the questionnaires. Details about the study protocol, questionnaire development, and sampling of GPs, have been published elsewhere [28–30].

The informed consent of participants was obtained, either written or verbal, depending on the mode of patient data collection (face to face in the practice in most countries) and legal requirements in each country. Ethical approval for the QUALICOPC study was acquired in accordance with the legal requirements in each country [31].

### Measurements

#### Dependent variables

To assess the patients’ perceptions of communication between GPs and medical specialists, we used the patients’ responses to the following statements:

- ‘When I am referred, my GP informs the medical specialist about my illness’;
- ‘After treatment by a medical specialist, my GP knows the results’.

Patients were asked whether they agreed with these statements. Possible answers to both questions were: ‘yes’; ‘no’; ‘don’t know’, and; ‘not applicable’. The questionnaire did not include a question on whether, or when, a patient had a referral from their GP to a medical specialist. We excluded patients who had never been referred (and therefore answered ‘not applicable’), and those who were not sure or not recently referred (and answered ‘don’t know’, because they could not remember). We only included ‘yes’ and ‘no’ answers, to ensure that data represented patients who remembered a referral to a medical specialist. We excluded a total of 14,283 respondents answering ‘don’t know’, and 3189 ‘not applicable’, for the first statement, and 9023 ‘don’t know’, and 2479 ‘not applicable’, for the second statement.

#### Independent variables

For the purpose of the analyses, we organised the independent variables of primary interest according to our hypotheses:

##### Rates of communication reported by GPs at the interface between primary and secondary care

To measure communication at the interface between primary and secondary care from the GPs’ perspectives, we used two questions from the questionnaire completed by GPs within the QUALICOPC study [29]. GPs were asked to indicate how often they send referral letters to medical specialists, and how often they receive feedback from the specialists: (Scaioli G, Schäfer W, Boerma W , Spreeuwenberg P, Schellevis FG, Groenewegen PP. Communication between general practitioners and medical specialists in the referral process: a cross sectional survey in 34 countries, submitted), [29).

- ‘To what extent do you use referral letters (including details on provisional diagnosis and possible test results) when you refer patients to a medical specialist?’
- ‘To what extent do medical specialists inform you after they have finished the treatment or diagnostics of your patients?’

Possible answers for the first question were: ‘for all patients whom I refer’; ‘for most patients whom I refer’; ‘for a minority of patients whom I refer’, and; ‘seldom or never’. Possible answers for the second question were: ‘Always, or nearly always’; ‘Usually’; ‘Occasionally’, and; ‘Seldom or Never’. The answers to both the above-mentioned questions were dichotomised as follows: ‘seldom or never’ versus the other three possible answers (Scaioli G, Schäfer W, Boerma W , Spreeuwenberg P, Schellevis FG, Groenewegen PP. Communication between general practitioners and medical specialists in the referral process: a cross sectional survey in 34 countries, submitted).

##### Personal continuity of care

We used measures of personal continuity of care at the country level and at the patient level. To estimate the level of personal continuity of care at the country level, we listed the countries in which it is mandatory for a patient to be registered to a specific GP or practice (‘list system in place’). For 30 countries (excluding Australia, Canada, New Zealand, and the former Yugoslav Republic of Macedonia), we derived these data from the PHAMEU (Primary Health Care Activity Monitor) study [32, 33]. Data from the remaining four countries were collected using the same methods as the PHAMEU study [22, 33]. To measure the level of personal continuity of care at the patient level, we asked the patients whether they have their own doctor, for instance a GP, whom they normally consult first with a health problem. Possible answers were: ‘Yes, the doctor I have just visited’; ‘Yes, but another doctor in this practice or centre’; ‘Yes, but another doctor from somewhere else’, and; ‘No, I do not have my own doctor’. We dichotomised the answers into ‘Yes’ and ‘No’.

##### Job satisfaction of GPs

We measured the job satisfaction of GPs through a continuous score derived from a combination of six statements from the questionnaire addressed to GPs within the QUALICOPC study. A higher score measures higher job satisfaction. The development of the score was described in detail in another paper (Schäfer WL, Van den Berg M, Groenewegen PP. The association between the workload of family physicians and patient experiences with care, submitted).

#### Confounders

We included the following potentially confounding factors in our analyses:

- Age and gender of GPs;
- Self-reported urbanisation of the practice location. Possible locations were ‘big (inner)city’, ‘suburbs’, ‘(small) town’, ‘mixed urban-rural’, and ‘rural’;
- Self-reported employment status of GPs (‘salaried’, ‘self-employed’, ‘mixed’);
- Age and gender of patients;
- Self-reported income of patients, compared to the average of the country (‘below average’, ‘around average’, ‘above average’);
- patients perception of their health status gained through answers to the question: ‘How would you describe your own health in general?’ (possible answers: ‘very good’, ‘good’, ‘fair’, ‘poor’); and,
- If patients had one or more self-reported longstanding diseases (such as high blood pressure, diabetes, depression, asthma or another longstanding condition).

### Statistical analysis

The distributions of answers to the two outcome statements: ‘When I am referred, my GP informs the medical specialist about my illness’, and: ‘After treatment by a medical specialist, my GP knows the results’, were calculated for each country. Next, multi-response logistic multilevel models were used, firstly, to calculate odds ratios in order to analyse the relationship between the dependent and independent variables. Then they were used to analyse the correlation between patients’ perceptions of GPs’ communication to medical specialists, firstly about their illness when they are referred, and, secondly, on receipt of results by GPs of treatment carried out by medical specialists. The country, GP, patient, and response levels were included in our models. We estimated an initial model that included only confounders. These were: age and gender of GPs; practice location; employment status of GPs; age and gender of patients; income of patients; perceived health status of patients; and presence of at least one longstanding disease. We applied three cumulative models, in which we added variables that assessed: 1) the rates of referral letters sent, and feedback communication received by GPs, as declared by GPs; 2) job satisfaction of GPs; 3) personal continuity of care at the patient and country level. We only present the final model in the results section as the odds ratios only change slightly over the subsequent models. For each of the two outcome variables, we calculated the percentage of reduction of variance in the final model at the country and GP levels. The level of statistical significance was set at *p* < 0.05. We used Stata version 13.0 (StataCorp. LP, College Station, USA) for the descriptive statistics, and MLwiN, version 2.25 (University of Bristol, Bristol, UK) for the multi-response logistic multilevel models.

## Results

Table 1 provides a summary of the sample characteristics of the patients, GPs, and countries included in the study.

**Table 1:** Characteristics of patients, primary care practices, GPs and countries included in the study

*Characteristics of patients (N = 60,762)*	
Gender (percentage female)	61.0
Age (mean ± SD^a^)	51.0 ± 17.4
Income (percentage, compared to the average of the country)	
Below average	30.8
Around average	56.9
Above average	12.3
Perceived health status (percentages)	
Poor	8.8
Fair	30.7
Good	46.2
Very good	14.3
Presence of at least one longstanding disease (percentage yes)	49.9
Patients with a personal doctor (percentage yes)	95.7
*Characteristics of primary care practices and GPs (N = 7183)*	
Gender of GPs (percentage female)	52.2
Age of GPs (mean ± SD^a^)	50.3 ± 9.6
Practice location (percentages)	
Big (inner)city	31.8
Suburbs or small town	34.6
Mixed urban-rural or rural	33.4
Employment status (percentages)	
Salaried	33.7
Self-employed	65.4
Mixed	0.9
Frequency of referral letters sent to medical specialists by GPs (percentage)	
For all the patients who they refer	64.9
For most patients who they refer	20.8
For a minority of patients who they refer	7.8
Seldom or never	6.5
Frequency of feedback received from medical specialists (percentage)	
Always, or almost always	38.1
Usually	35.8
Occasionally	14.4
Seldom or never	11.7
Score of job satisfaction of GPs, range 1–4 (mean ± SD^a^)	2.5 ± 0.5
*Characteristics of countries (N = 34)*	
List system in place (percentage yes)	52.9

^a^*SD* Standard Deviation

### Rates of perceived communication

**Fig. 2:**
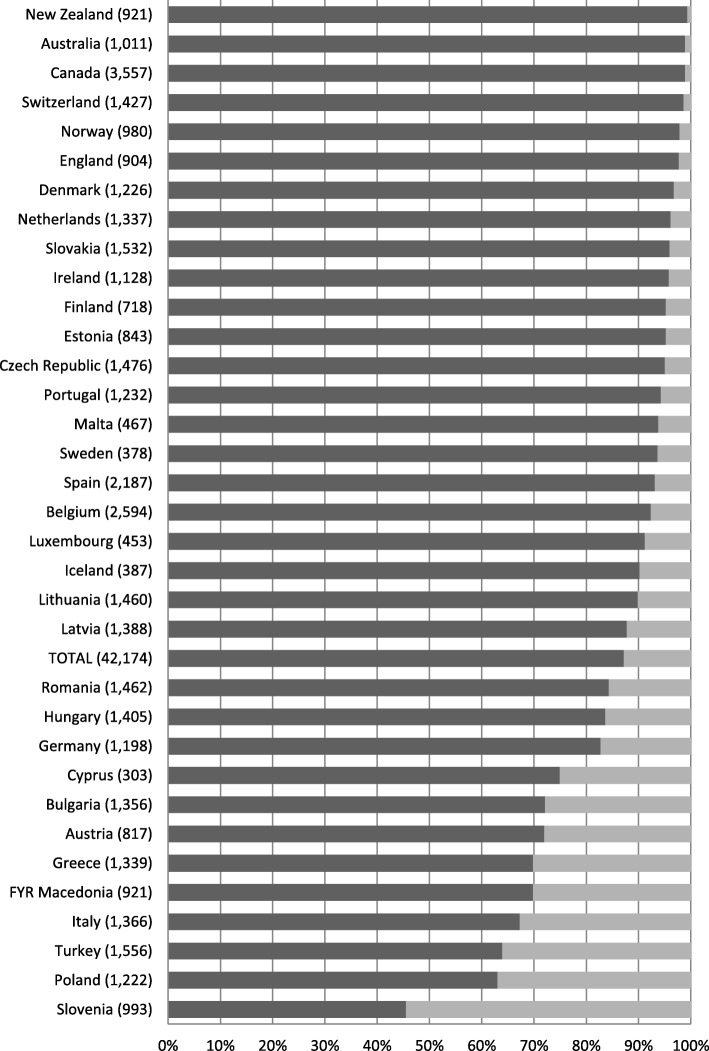
Percentages of patients who agreed, and disagreed, with the statement: “When I am referred, my GP informs the medical specialist about my illness”, by country, numbers between brackets

**Fig. 3:**
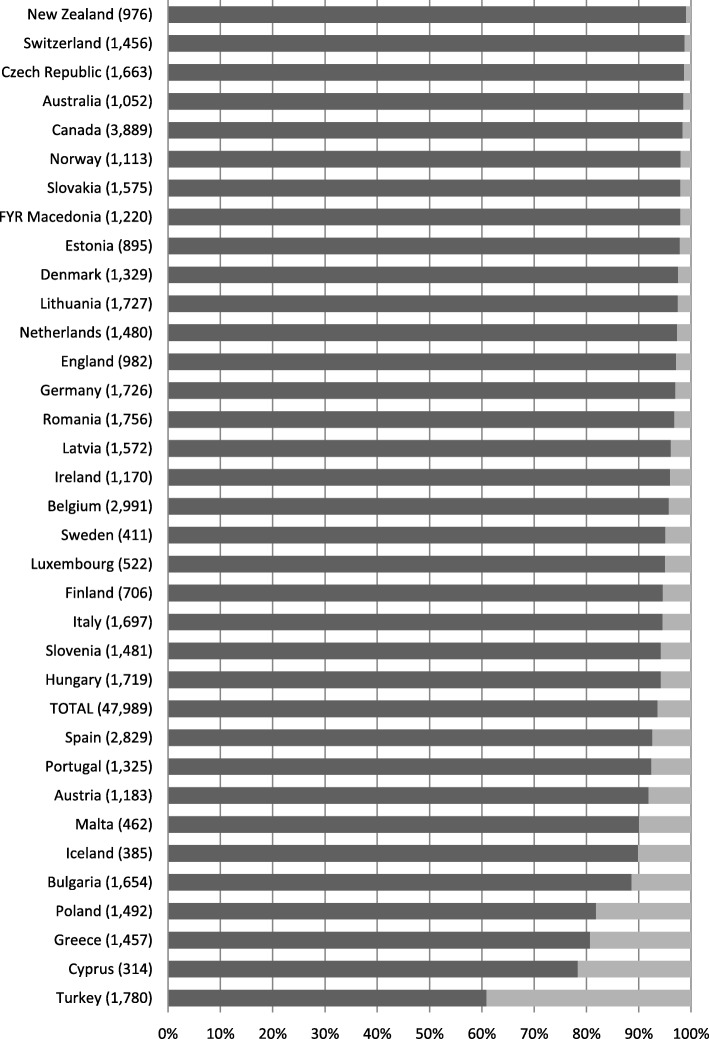
Percentages of patients who agreed, and disagreed, with the statement: “After treatment by a medical specialist, my GP knows the results”, by country, numbers between brackets

Figures 2 and 3 show the distributions of answers for the two outcome statements by country. The majority of the patients surveyed agreed with the statement: ‘When I am referred, my GP informs the medical specialist about my illness’. However, we found large differences across countries. In New Zealand, the percentage of patients who agreed with this statement was 99.4%, and in Slovenia this was only 45.5%. The majority of the patients also reported that they thought that their GP would know the results after a treatment by a medical specialist. Again, large differences between countries were found, varying from 99.1% for New Zealand and 60.8% for Turkey.

### Associated factors

Tables 2 and 3 show the results of the final multi-response logistic multilevel model. Patients were less likely to indicate that their GP informs the medical specialist about their illness upon referral, if their GPs have stated that they send referral letters ‘seldom or never’ as opposed to those GPs who send referral letters ‘for all patients’, ‘for most patients’, or ‘for a minority of patients’. Patients are more likely to indicate that their GP informs the medical specialist, if they have a personal doctor and when their GP has a higher job satisfaction, although the latter does not have a significant association (Table 2). The variance at the country level and at the GP level is high. The intraclass correlations (ICCs) of the final model are 26 and 27% at the country and a GP level, respectively. The variances at the country and the GP level of the final model only slightly decrease compared to the variance of the null model which only includes the confounders. This final model provides a reduction of 4.2% of the variance at the country level, and only 0.7% at the GP level (Table 2).

**Table 2:** Factors related to rates of patients’ perception of communication from GPs to medical specialists about their illness when they are referred (multi-response logistic multilevel analysis)

N_countries_ = 34 N_GPs_ = 6519 N_patients_ = 38,432	Odds Ratio^a^	(CI)
*Patient level*		
The patient has a personal doctor	1.99	(1.67, 2.36)
*GP level*		
Frequency of referral letters sent to medical specialists by GPs (ref. more than ‘seldom or never’)		
Seldom or never	0.75	(0.64, 0.87)
Score of job satisfaction of GPs	1.1	(1.00, 1.22)
*Country level*		
List system in place	0.76	(0.41, 1.41)
Variance country level	1.79	
Variance GP level	1.88	
Percentage of reduction of variance (Country level)^b^	4.2%	
Percentage of reduction of variance (GP level)^c^	0.7%	
Intraclass correlation (ICC) country level	26%	
Intraclass correlation (ICC) GP level	27%	

*CI* 95% confidence interval

^a^adjusted also for age and gender of GPs, practice location, employment status of GPs, age and gender of patients, income of patients, perceived health status of patients and presence of at least one longstanding disease

^b^this percentage was calculated by using the variance at a country level of the initial model (variance initial model =1.88)

^c^this percentage was calculated by using the variance at a GP level of the initial model (variance initial model =1.90)

**Table 3:** Factors related to patients’ perception of receipt, by GPs, of results of treatment carried out by medical specialists in 34 countries (multi-response logistic multilevel analysis)

N_i_ = 34 countries N_j_ = 6529 GPs N_k_ = 43,802 patients	Odds Ratio^a^	(CI)
*patient level*		
The patient has a personal doctor	2.30	(1.91, 2.75)
*GP level*		
Frequency of referral letters sent to medical specialists by GPs (ref. more than ‘seldom or never’)		
Seldom or never	0.83	(0.72, 0.95)
Score of job satisfaction of GPs	1.11	(1.00, 1.24)
*Country level*		
List system in place	1.31	(0.85, 2.03)
Variance Country level (SE)	1.03	
Variance GP level (SE)	1.71	
Percentage of reduction of variance (Country level)^b^	12%	
Percentage of reduction of variance (GP level)^c^	0.5%	
Intraclass correlation (ICC) country level	17%	
Intraclass correlation (ICC) GP level	28%	

*CI* 95% confidence interval

^a^adjusted also for age and gender of GPs, practice location, employment status of GPs, age and gender of patients, income of patients, perceived health status of patients, and presence of at least one longstanding disease

^b^this percentage was calculated by using the variance at a country level of the empty model (variance empty model =1.18)

^c^this percentage was calculated by using the variance at a country level of the empty model (variance empty model =1.72)

Patients with GPs who indicate they ‘seldom or never’ receive feedback from medical specialists are less likely to state that their GPs are informed about the results of the specialists’ treatments. On the other hand, patients with a personal doctor have a higher likelihood of stating that their GP knows about the results of the specialist’s treatment. Higher job satisfaction among GPs is associated with higher perceived rates of feedback received by GPs, although, here too, the association is not significant. The ICC of the final model at the country level is 17%, while the ICC at the GP level is 28%. The variance only decreased slightly from the variance of the null model applying only confounders. For this outcome measure, the final model provides a reduction of variance of 12% at the country level and 0.5% at the GP level.

There is a strong correlation at the country level and at the GP level between our two outcome measures (the correlation coefficients are 0.65 and 0.46 respectively). The correlation coefficients only change slightly after adjusting for all the covariates (0.69 at the country level and 0.46 at the GP level) (data not shown).

## Discussion

This study aimed to investigate factors correlating to the rates of communication perceived by the patient at the interface between primary and secondary care. We found that these rates correlate positively with the personal continuity of care experienced, and with the rates of communication at the interface between primary and secondary care, as reported by GPs.

In line with our first hypothesis, we found that there is a positive association between the communication perceived by the patient at the interface between primary and secondary care, and the rates of sending referral letters and receiving feedback communication reported by GPs. This means that when a GP sends a referral letter to and/or receives feedback from a medical specialist, the patients are usually aware of that. This is easily understandable when the patient is the carrier of the paper letter, that is when the GP and/or a medical specialist gives the letter directly to the patient. But it is less obvious when the GP and the medical specialist communicate through electronic means. However, a previous study found that the use of electronic systems to communicate did not affect the perception by patients of this communication [34].

Our second hypothesis was partially confirmed. Indeed, patients with a personal doctor were more likely to state that their GPs send referral letters to medical specialists and receive feedback. This means that higher ‘personal continuity of care’ is positively associated with patients’ perceived ‘cross-boundary continuity of care’ [7, 8]. Thus, this study demonstrated that different dimensions of continuity of care are interrelated. This is in line with findings from previous studies on this topic [4, 35]. However, the presence of a list system in a country is not significantly associated with higher rates of communication at the interface between primary and secondary care as perceived by the patient. This indicates that having a list system in place, and having a personal doctor are potentially different dimensions of ‘personal continuity of care’.

Unexpectedly, and in contrast with our third hypothesis, job satisfaction among GPs is not significantly correlated with our two main outcome measures. In a previous paper, using data from the same QUALICOPC study, Schäfer et al. demonstrated that high job satisfaction among GPs is related positively to patients’ perceived quality of care (Schäfer WL, Van den Berg M, Groenewegen PP. The association between the workload of family physicians and patient experiences with care, submitted). Moreover, a recent study demonstrated that subjective workload might affect communication between GPs and medical specialists negatively during referrals (Scaioli G, Schäfer W, Boerma W , Spreeuwenberg P, Schellevis FG, Groenewegen PP. Communication between general practitioners and medical specialists in the referral process: a cross sectional survey in 34 countries, submitted).

The results of the multi-response analysis demonstrated that there is a strong correlation at country level between the answers to the two outcome statements. As there is a positive association between a patient’s perception of communication and rates of communication reported by GPs, this means that there are countries that perform better in the area of communication between GPs and medical specialists in both directions. Notably, the three non-European countries, together with Switzerland, are in the top five countries in both outcome statements. With the exception of Switzerland, these countries have a strong primary care system and, in particular, in the area of continuity of care, there is little room for improvement, according to patients [21]. The countries at the bottom of the distribution differ for both outcome statements. However, they include some of the weaker primary care systems, such as Poland, Greece, Cyprus and Turkey. This shows, too, in the potential for improvement suggested by patients.

It should be noted that the final explanatory model provides only a relatively small reduction of variance at the country and at the GP levels for the two outcomes considered. This means that there are other factors that potentially influence patients’ perception of communication at the interface between primary and secondary care which were not measured in our study. Examples of these might include the implementation and use of personal health records, [36, 37] and/or features of medical specialists and their practice organisation.

As patients positive perceptions of communication between primary care physicians and medical specialists improve health outcomes, [7, 14] the results of this study could be useful for decision-makers to understand the benefits of modifying simple features of the organisation of primary care. For example, this could include stimulating patients to have a personal doctor, thus increasing the rates of patients’ perception of communication. Furthermore, the rates of patients’ perception of communication at the interface with secondary care are correlated with those of the GPs’ reported rates of communication. Therefore, policies aimed at stimulating communication between GPs and medical specialists could also improve patients’ perception of this communication (Scaioli G, Schäfer W, Boerma W , Spreeuwenberg P, Schellevis FG, Groenewegen PP. Communication between general practitioners and medical specialists in the referral process: a cross sectional survey in 34 countries, submitted) and thereby the cross-boundary continuity of care [38].

### Strengths and limitations

The main strength is the number of countries studied. Patients from 34 countries were surveyed, and this gave us the opportunity to investigate whether differences in health care systems were related to communication as perceived by the patient at the interface between primary and secondary care. Moreover, we were able to assess whether the characteristics of GPs and patients affect the perception of patients about communication at this interface, independent of the national health care system. The use of multilevel regression analysis is another strength of this study. Multilevel analysis allowed us to examine, at the same time, the associations with variables at the group and individual levels, in order to account for the dependence of observations within groups, and to examine both variation within individuals and groups [39].

A limitation of this study is that it is only patients who visited a GP who were included. Therefore, the sample is not representative of the general population of each country. Moreover, in several countries, patients tend to visit a medical specialist without consulting a GP first. Our sample does not represent this group of patients. In our analyses, we also excluded all patients who answered, “don’t know”, to the two outcome questions about communication between GPs and medical specialists. These patients were excluded however, because they may have been referred some time ago and/or don’t remember what happened around a specific referral. Another limitation is that we could not assess whether the characteristics of medical specialists are associated with the patients’ perspective on communication at the interface between primary and secondary care. Finally, the study does not include data about how patients perceived the quality of this communication. The Qualicopc study was designed covering a broad range of topics and not as a study into referral processes and communication. We were therefore restricted in the nature and amount of questions we could use. The patient perceived quality and the referral process more broadly should be further explored in international research.

## Conclusion

This study revealed large differences among countries in patients’ perceptions of communication at the interface between primary and secondary care, However, personal continuity of care can improve this perception, independent of the health care system in which this occurs. The results also indicate the need for further studies to investigate other factors potentially associated with patients’ perception of communication at the interface between primary and secondary care.

## Data Availability

The datasets used and/or analysed during the current study are available from the corresponding author on reasonable request.

## Abbreviations

GP: General practitioner
ICC: Intraclass correlations
PHAMEU: Primary Health Care Activity Monitor
QUALICOPC: Quality and costs of primary care
